# Progress report on the first sub-Saharan Africa trial of newer versus older antihypertensive drugs in native black patients

**DOI:** 10.1186/1745-6215-13-59

**Published:** 2012-05-17

**Authors:** Augustine N Odili, Birinus Ezeala-Adikaibe, Mouhamadou B Ndiaye, Benedict C Anisiuba, Marius M Kamdem, Chinwuba K Ijoma, Joseph Kaptue, Hilaire J Boombhi, Philip M Kolo, Elvis N Shu, Lutgarde Thijs, Jan A Staessen, Babatunde A Omotoso, Samuel Kingue, Serigne A Ba, Daniel Lemogoum, Jean-René M’Buyamba-Kabangu, Ifeoma I Ulasi

**Affiliations:** 1 Studies Coordinating Centre, Division of Hypertension and Cardiovascular Rehabilitation, Department of Cardiovascular Diseases, University of Leuven Campus Sint Rafaël, Kapucijnenvoer 35, Block D, Box 7001, Leuven BE-3000, Belgium; 2 Department of Internal Medicine, College of Health Science, University of Abuja, Abuja, Nigeria; 3 Department of Medicine, College of Medicine, University of Nigeria Teaching Hospital, Enugu, Nigeria; 4 Douala Cardiovascular Research Institute, Douala School of Medicine, Douala, Cameroon; 5 Centre Hospitalier National Aristide Le Dantec, Dakar, Senegal; 6 Yaoundé General Hospital, Yaoundé, Cameroon; 7 Department of Medicine, University of Ilorin Teaching Hospital, Ilorin, Nigeria; 8 Department of Pharmacology and Therapeutics, College of Medicine, University of Nigeria Teaching Hospital, Enugu, Nigeria; 9 Department of Epidemiology, Maastricht University, Maastricht, Netherlands; 10 Hypertension Unit, Department of Internal Medicine, University of Kinshasa Hospital, Kinshasa, Democratic Republic of Congo

**Keywords:** Antihypertensive therapy, Health policy and outcome research, Randomized clinical trial, Special populations

## Abstract

**Background:**

The epidemic surge in hypertension in sub-Saharan Africa is not matched by clinical trials of antihypertensive agents in Black patients recruited in this area of the world. We mounted the Newer versus Older Antihypertensive agents in African Hypertensive patients (NOAAH) trial to compare, in native African patients, a single-pill combination of newer drugs, not involving a diuretic, with a combination of older drugs including a diuretic.

**Methods:**

Patients aged 30 to 69 years with uncomplicated hypertension (140 to 179/90 to 109 mmHg) and ≤2 associated risk factors are eligible. After a four week run-in period off treatment, 180 patients have to be randomized to once daily bisoprolol/hydrochlorothiazide 5/6.25 mg (R) or amlodipine/valsartan 5/160 mg (E). To attain blood pressure <140/<90 mmHg during six months, the doses of bisoprolol and amlodipine should be increased to 10 mg/day with the possible addition of up to 2 g/day α-methyldopa.

**Results:**

At the time of writing of this progress report, of 206 patients enrolled in the run-in period, 140 had been randomized. At randomization, the R and E groups were similar (*P* ≥ 0.11) with respect to mean age (50.7 years), body mass index (28.2 kg/m^2^), blood pressure (153.9/91.5 mmHg) and the proportions of women (53.6%) and treatment naïve patients (72.7%). After randomization, in the R and E groups combined, blood pressure dropped by 18.2/10.1 mmHg, 19.4/11.2 mmHg, 22.4/12.2 mmHg and 25.8/15.2 mmHg at weeks two (n = 122), four (n = 109), eight (n = 57), and 12 (n = 49), respectively. The control rate was >65% already at two weeks. At 12 weeks, 12 patients (24.5%) had progressed to the higher dose of R or E and/or had α-methyldopa added. Cohort analyses of 49 patients up to 12 weeks were confirmatory. Only two patients dropped out of the study.

**Conclusions:**

NOAAH (NCT01030458) demonstrated that blood pressure control can be achieved fast in Black patients born and living in Africa with a simple regimen consisting of a single-pill combination of two antihypertensive agents. NOAAH proves that randomized clinical trials of cardiovascular drugs in the indigenous populations of sub-Saharan Africa are feasible.

## Background

Sub-Saharan Africa faces an unprecedented epidemic of cardiovascular disease [1-3]. Hypertension is the main driver of cardiovascular complications. Whereas high blood pressure almost did not exist in Black Africa in the first half of the twentieth century [4], hypertension now affects between 30% and 60% of African Blacks [5]. Lowering blood pressure and controlling hypertension is key to cardiovascular disease prevention.

Practice guidelines issued by the World Health Organization and the International Society of Hypertension (WHO/ISH) in 2003 recommended that a low dose diuretic should be considered as the first choice of therapy on the basis of comparative trial data, availability and cost [6]. Several randomized clinical trials published since 2003, proved that a combination of newer antihypertensive agents, compared with older antihypertensive agents [7] or combinations including a diuretic [8], lowered blood pressure more with fewer metabolic side-effects and, more importantly, further reduced the risk of cardiovascular complications. Because trials of antihypertensive drug treatment are scarce in Black patients born and living in Africa, we recently mounted the Newer versus Older Antihypertensive agents in African Hypertensive Patients (NOAAH) trial (nct01030458) to compare the efficacy of single-pill combinations of newer versus older antihypertensive agents [9]. This NOAAH progress report describes recruitment and baseline characteristics of the randomized patients and the blood pressure control after randomization as part of the ongoing efforts invested in quality assurance and control and in providing feedback to the clinical investigators and building capacity to run randomized clinical trials of antihypertensive drugs in this part of the world.

## Methods

The detailed protocol of the NOAAH trial has been published elsewhere [9]. NOAAH is an investigator-led trial conducted at seven sub-Saharan centers, six of which enrolled patients. NOAAH complies with the guidelines for good clinical practice outlined in the Helsinki declaration [10]. Each participating center received approval from the local Institutional Review Board and, if applicable, from National Regulatory Authorities. The Sponsor (Hypertension Unit, University of Kinshasa Hospital, Democratic Republic of Congo) obtained ethical approval from the Ethics Committee of the Faculty of Medicine.

### Selection of patients

Eligible patients are treatment naïve or previously treated patients of either sex with grade-1 or grade-2 hypertension [11]. Age must range from 30 to 69 years. Women with childbearing potential must apply effective contraception. Hypertension must be uncomplicated with a maximum of two additional risk factors, as defined in the 2007 guidelines [11] of the European Societies of Cardiology and Hypertension (ESC/ESH). A history of cardiovascular disease, electrocardiographic left ventricular hypertrophy and diabetes mellitus were exclusion criteria. The blood pressure determining eligibility is the average of three consecutive readings obtained with patients in the seated position after at least four weeks follow-up off antihypertensive treatment. Blood pressure must range from 140 to 179 mmHg systolic and from 90 to 109 mmHg diastolic. The standing systolic blood pressure, the average of three consecutive readings obtained immediately after the sitting measurements, must be at least 110 mmHg.

The presence of three or more risk factors, a history of cardiovascular disease or diabetes mellitus [12] are exclusion criteria. Previously treated patients should not have a compelling indication according to the ESC/ESH guidelines [11] to continue antihypertensive drug treatment and should not take more than a single drug or one single-pill combination at the enrollment visit. Other exclusion criteria include: atrial fibrillation; electrocardiographic left ventricular hypertrophy with strain pattern or electrocardiographic left ventricular hypertrophy defined as a Sokolow-Lyon index larger than 38 mm (3.8 mV) or a Cornell voltage × duration QRS index larger than 2,440 mm × msec [13-15]; renal dysfunction defined as a serum creatinine concentration higher than 1.4 mg/dL in women or 1.5 mg/dL in men [16]; proteinuria or hematuria as detected by a semi-quantitative dipstick test; severe non-cardiovascular disorders; psychiatric illness; and substance abuse.

### Design

The study starts with a screening visit, at which informed consent is obtained, followed by a run-in period with two subsequent visits at two-week intervals. Investigators keep a log of the patients screened for enrollment. Patients entering the run-in period provide informed consent. Literate patients sign and date the informed consent form. Illiterate patients provide a fingerprint in the presence of an independent witness, who also has to sign the consent form. At the screening visit, antihypertensive drug treatment is discontinued and lifestyle changes are recommended and reinforced at the subsequent run-in visits.

Study forms are emailed to the Studies Coordinating Center (SCC, Leuven, Belgium). After checking the eligibility criteria and the quality and completeness of the run-in forms, SCC randomizes patients in a 1:1 proportion, using permuted blocks of four consecutive patients within each center. Patients are randomly allocated to a single-pill combination of 6.25 mg hydrochlorothiazide plus 5 mg bisoprolol to be up-titrated to 6.25 mg hydrochlorothiazide plus 10 mg bisoprolol (Lodoz®, Merck Serono) in the reference group or to the combination of amlodipine 5 mg plus valsartan 160 mg to be up-titrated to amlodipine 10 mg with valsartan 160 mg (Exforge®, Novartis) in the experimental group. In the two treatment groups, α-methyldopa (Aldomet®) up to 2 g per day is used, if the blood pressure remains uncontrolled on the maximally tolerated dose of the randomized medication. After randomization, patients are followed up for six months with visits scheduled after two weeks and four weeks and monthly thereafter until the end of the study.

The primary outcome is the between-group difference in the change in systolic blood pressure achieved on randomized treatment and measured with the patient in the seated position [9]. Secondary outcomes are the time interval required after randomization to achieve blood pressure control defined as a level below 140 mmHg systolic and 90 mmHg diastolic, the incidence of adverse events (including metabolic side effects), and the adherence to the study medication and drop-out rate [9]. To demonstrate a 5-mmHg between-group difference in the achieved systolic blood pressure (SD, 12 mmHg) with a 2-sided *P*-value of 0.01 and 90% power, 180 randomized patients, 90 per treatment group, are required [9].

### Measurements

Blood pressure is measured by means of validated [17] oscillometric OMRON *705IT* recorders (OMRON Healthcare Europe BV, Nieuwegein, Netherlands) according to the ESC/ESH guideline [18]. Use of automated devices allows blinded assessment of the blood pressure endpoints in an open trial, in which neither patients nor investigators are blinded with regard to treatment allocation. A standard cuff with an inflatable bladder of 22 × 12 cm will be used if arm circumference is less than 32 cm and cuffs with a 35 × 15 cm bladder on larger arms. Investigators record standard 12-lead electrocardiograms by means of the paperless Cardiax device (http://www.rdsm.eu/cardiax.html), interfaced with a computer. Biochemical measurements include hemoglobin, hematocrit, red and white blood cell counts, serum potassium, creatinine and total cholesterol, blood glucose, and a dipstick test on fresh urine to detect glucosuria, proteinuria and hematuria. To assess side effects, we used a simple two-page questionnaire, which we validated in previous studies [19,20].

### Drug accountability

SCC shipped Lodoz®, Exforge® and Aldomet® to the recruiting clinical sites. To track the flow of medication, each box of drugs carries a unique identification number. Patients should return unused medication at the next visit. Investigators count the number of unused pills. Patients are classified as compliant, if they took at least 80% of the prescribed study medication and if they did not miss any dose on the days of the clinic visits. Whenever drugs are dispensed or recouped investigators complete a drug accountability form to be forwarded to SCC.

### Database management and statistical analysis

SCC developed the case report forms as interactive PDF forms, which investigators complete at the clinical sites and print for the local patient files. XML files exported from the PDFs are sent to SCC as email attachments. After quality control and addition of the codes for symptoms, diseases and concurrent medications at SCC, the XML files are directly imported into the SAS database, using the SAS XML Mapper, version 9.2.

For database management and statistical analyses, we used SAS 9.3 (SAS Institute, NC, USA). In this report, we compared means and proportions by Student’s *t* test and χ^2^ statistic, respectively. Statistical significance is a two-sided *P* value of 0.05.

## Results

**Figure 1:**
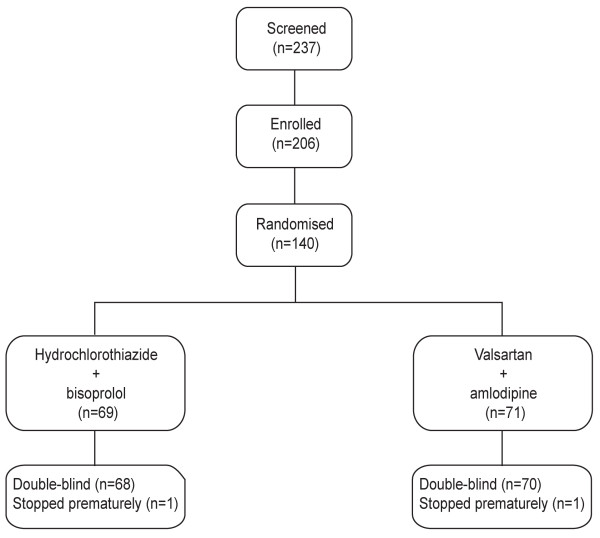
Trial profile based on data available on 14 April 2011.

**Figure 2:**
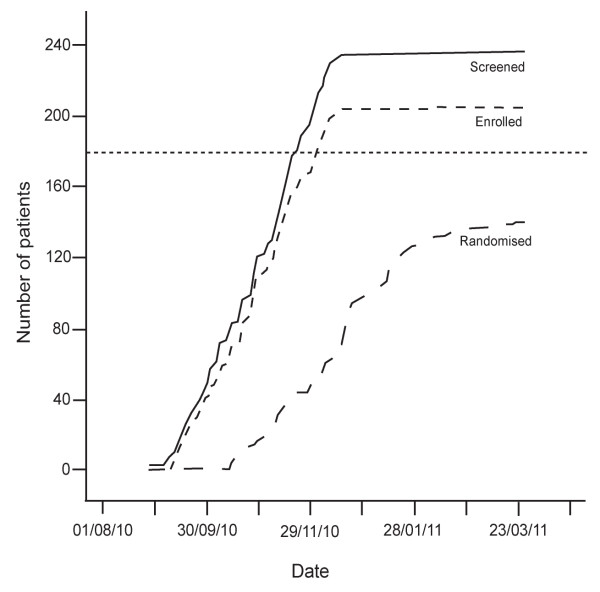
**Number of patients screened, enrolled and randomized on 14 April 2011.** The horizontal dotted line indicates the number of patients to be randomized.

This report is based on the data available at SCC on 14 April 2011. At this point (Figure 1), 237 patients had been screened, 206 had been enrolled into the four week run in period, and 69 and 71 had been randomized to control (hydrochlorothiazide plus bisoprolol) or experimental treatment (valsartan plus amlodipine), respectively. Figure 2 gives the number of patients screened, enrolled in the run-in phase, and randomized. The maximum follow up varied, because the patients had been enrolled over several months starting on 1 September 2010. The number of patients with a follow-up of two weeks and one, two, three and four months was 122, 109, 57, 49 and 24, respectively; the proportion of adherent patients at these visits was 89.6%, 76.7%, 76.4%, 73.2% and 83.3%, respectively.

### Patient characteristics at baseline

In all 140 patients, age at randomization averaged 50.7 ± 8.9 years, ranging from 30.5 to 68.9 years. The untreated blood pressure measured in the sitting position at the end of the run-in period was 153.9 ± 11.3 mmHg systolic and 91.5 ± 9.9 mmHg diastolic; the corresponding measurements in the standing position were 154.5 ± 13.2 mmHg and 97.3 ± .9.9 mmHg, respectively. Of the 140 randomized patients, 75 (53.6%) were women.

Among the 75 women, average values of the anthropometric measurements were 162.6 ± 6.9 cm for height, 76.7 ± 13.6 kg for body weight, 29.0 ± 5.1 kg/m^2^ for body mass index, and 92.2 ± 13.1 cm for waist circumference; the corresponding values in the 65 men were 172.0 ± 7.2 cm, 81.3 ± 15.3 kg, 27.4 ± 4.4 kg/m^2^, and 95.3 ± 12.2 cm, respectively.

Table 1 shows that the patients randomized to the control and experimental treatments had similar characteristics (*P* ≥ 0.11). Of the 140 participants, four (2.9%; 0 women and four men) were current smokers, none snuffed tobacco, and 44 (31.6%; 12 women and 32 men) reported regular drinking. In drinkers, the median alcohol consumption was 7.1 g per day (interquartile range, 2.9 to 11.4).

**Table 1:** 

**Characteristic**	**Older drugs**	**Newer drugs**	**Difference (95% CI)**	***P***
Number	69	71		
Anthropometrics				
Women, n [%]	33 (47.8)	42 (59.2)		0.23
Age, years	50.5 ± 8.6	50.9 ± 9.2	0.4 (−2.6 to 3.4)	0.78
Weight, kg	80.8 ± 14.6	76.9 ± 14.7	−4.0 (−8.9 to 0.9)	0.11
Height, cm	167.8 ± 7.8	166.4 ± 9.0	−1.2 (−4.1 to 1.6)	0.39
Body mass index, kg/m^2^	28.7 ± 4.6	27.8 ± 5.1	−0.9 (−2.5 to 0.8)	0.30
Waist circumference, cm	94.5 ± 13.9	92.9 ± 11.5	−1.6 (−5.9 to 2.7)	0.47
Sitting blood pressure				
Systolic, mm Hg	154.2 ± 11.0	153.7 ± 11.8	−0.5 (−4.3 to 3.3)	0.80
Diastolic, mm Hg	92.2 ± 9.4	90.8 ± 10.3	−1.4 (−4.7 to 1.9)	0.41
Heart rate, beats/minute	74.0 ± 9.5	73.1 ± 10.3	−0.9 (−4.2 to 2.4)	0.59
Standing blood pressure				
Systolic, mm Hg	154.9 ± 13.8	154.1 ± 12.8	−0.7 (−5.2 to 3.7)	0.74
Diastolic, mm Hg	97.1 ± 9.5	97.5 ± 10.2	0.4 (−2.9 to 3.7)	0.82
Heart rate, beats/minute	81.6 ± 9.5	80.6 ± 10.3	−1.0 (−4.7 to 2.7)	0.60
Lifestyle				
Past smokers, n [%]	9 (13.2)	13 (18.3)		0.48
Current smokers, n [%]	3 (4.4 )	1 (1.4 )		0.35
Sniffing tobacco, n [%]	0 (0.0 )	0 (0.0 )		1.00
Current drinkers, n [%]	22 (32.4)	22 (31.0)		1.00
Blood measurements				
Hemoglobin, mg/dL	12.9 ± 1.7	12.8 ± 1.7	−0.1 (−0.7 to 0.5)	0.74
Hematocrit, %	39.0 ± 5.0	38.8 ± 5.7	−0.2 (−2.0 to 1.7)	0.86
Glucose, mmol/L	5.1 ± 0.9	4.9 ± 0.7	−0.2 (−0.4 to 0.1)	0.19
Cholesterol, mmol/L	4.9 ± 1.1	4.8 ± 1.2	−0.0 (−0.4 to 0.4)	0.86
Creatinine, μmol/L	86.1 ± 20.1	90.9 ± 34.5	4.8 (−4.7 to 14.3)	0.32
ECG measurements				
Sokolow-Lyon index, mm	28.1 ± 7.7	29.8 ± 7.2	1.7 (−0.8 to 4.2)	0.18
Cornell index, mm × msec	1818 ± 836	1771 ± 594	−48 (−290 to 194)	0.70

Values are mean ± SD, number of participants (%), or the difference between the two groups (95% confidence interval). Older and newer drugs refer to the single-pill combinations of hydrochlorothiazide plus bisoprolol or valsartan plus amlodipine, respectively. The blood pressure measurements are averages of three consecutive readings in each participant.

The number of participants with measurements of hemoglobin, hematocrit, glucose, cholesterol and creatinine are 62, 67, 67, 69 and 68 in the older drug group and 65, 68, 71, 68 and 71 in the newer-drug group. CI, confidence interval; ECG, electrocardiogram; n, number; SD, standard deviation.

### Follow-up after randomization

In the two treatment groups combined, at 12 weeks of follow-up, only ten patients (20.4%) had progressed to the higher dose of the reference or the experimental treatment of whom four had α-methyldopa added. In an additional two patients α-methyldopa was added to the lower dose of the study medication. None of the patients had crossed over.

**Figure 3:**
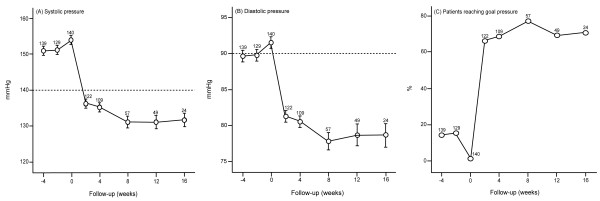
**Systolic blood pressure (A), diastolic blood pressure (B) and the proportion of patients controlled (C) during the course of the trial.** For this analysis the two treatment groups were combined and all available data were used. Blood pressure control is a level below 140 mmHg systolic and 90 mmHg diastolic.

**Figure 4:**
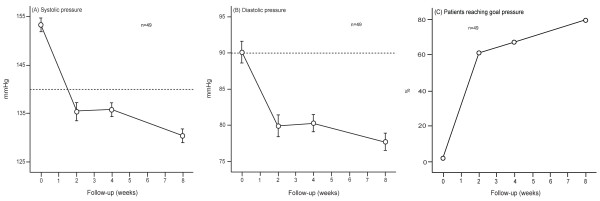
**Systolic blood pressure (A), diastolic blood pressure (B) and the proportion of patients controlled (C) during the course of the trial.** For this cohort analysis the two treatment groups are combined and the number of patients was constant in each of the data points (n = 49). Blood pressure control is a level below 140 mmHg systolic and 90 mmHg diastolic.

Table 2 lists the blood pressure levels at various lengths of follow-up. Systolic and diastolic pressures decreased (*P* < 0.001) after randomization by 18.2 and 10.1 mmHg at two weeks (n = 122); 19.4 and 11.2 mmHg at one month (n = 109); 22.4 and 12.2 mmHg at two months (n = 57); and 25.8 and 15.2 mmHg at three months (n = 49). On average, the reduction in blood pressure from randomization to the last available visit in 129 patients was 22.1 mmHg systolic and 12.4 mmHg diastolic (Table 2 and Figure 3). A cohort analysis of 49 patients, who had blood pressure measurements at each time point from randomization up to two months was confirmatory (Figure 4). Already starting from the second week after randomization, the control rates of hypertension were over 65%.

**Table 2:** Changes in blood pressure from baseline to follow-up.

**Follow-up visit**	**Baseline**	**Follow-up**	**Decrease (95% confidence interval )**
2 weeks (n = 122)			
Systolic pressure, mm Hg	154.4 ± 10.9	136.3 ± 14.2	18.2 (20.6 to 15.7)
Diastolic pressure, mm Hg	91.4 ± 10.3	81.3 ± 10.4	10.1 (11.8 to 8.4)
4 weeks (n = 109)			
Systolic pressure, mm Hg	154.7 ± 10.9	135.3 ± 13.6	19.4 (22.1 to 16.7)
Diastolic pressure, mm Hg	91.8 ± 10.2	80.6 ± 9.3	11.2 (13.0 to 9.5)
8 weeks (n = 57)			
Systolic pressure, mm Hg	153.5 ± 10.3	131.1 ± 11.8	22.4 (26.0 to 18.7)
Diastolic pressure, mm Hg	90.0 ± 9.3	77.8 ± 9.0	12.2 (14.7 to 9.6)
12 weeks (n = 49)			
Systolic pressure, mm Hg	156.9 ± 11.0	131.1 ± 14.3	25.8 (30.1 to 21.5)
Diastolic pressure, mm Hg	93.9 ± 9.1	78.7 ± 11.4	15.2 (18.1 to 12.2)
Last visit (n = 129)^a^			
Systolic pressure, mm Hg	154.4 ± 10.9	132.3 ± 14.4	22.1 (24.7 to 19.5)
Diastolic pressure, mm Hg	91.4 ± 10.1	79.0 ± 10.0	12.4 (14.2 to 10.7)

All changes in blood pressure were significant (*P* < 0.001).^a^Seven patients did not have a two-week visit. n, number.

Side effects led to premature termination of the trial in two patients: insomnia, asthenia and hot flushes in one patient and bilateral leg edema in the other patient. In addition, in three patients the study medication was adjusted because of heartburn, tiredness and diarrhea.

## Discussion

NOAAH is the first multicenter clinical trial on antihypertensive therapies that is exclusively running in sub-Saharan Africa. The underlying hypothesis is that in Blacks born and living in Africa the combination of newer agents will be more effective in blood pressure lowering and in achieving blood pressure control with fewer side-effects than the combination of older drugs. The trial is only running in countries in which the randomized medications are marketed and available to patients after they leave the trial. If our assumptions hold true, they challenge the current recommendation of the WHO/ISH [6] to start antihypertensive treatment with diuretics in Black hypertensive patients. This progress report confirms that the number of patients randomized in NOAAH reached approximately 80% of the projected sample size. With the current rate of recruitment and randomization, the final report will be available for publication in 2012.

Control of blood pressure to recommended target levels below 140 mmHg systolic and 90 mmHg diastolic remains a major challenge in Africa [21,22], Europe [23] and the United States [24]. In the ongoing NOAAH trial, within two weeks of randomization, the average blood pressure fell from 153.9 to 136.3 mmHg systolic and from 91.5 to 81.3 mmHg diastolic, while over 65% of the patients reached blood pressure control. Because NOAAH does not include an untreated control group, part of the blood pressure reduction might include placebo effects or habituation of the patients to the medical environment and the clinical investigators. Because blood pressure measurement is automated in NOAAH, the primary endpoint is measured free of observer bias.

Lowering blood pressure [25-28] and early blood pressure control [7,29] are essential for the prevention of cardiovascular complications. NOAAH will generate information that will be helpful in addressing the epidemic of cardiovascular disease in sub-Saharan Africa [30]. For instance, we recently assessed the frequency and determinants of in-hospital mortality among patients admitted for hypertension to two city hospitals in Mbuji Mayi, Congo [31]. Among 401 consecutive patients (mean age, 54.3 years; 129 women), 89 (22.2%) died over a median follow-up of 15 days. The multivariable-adjusted probability of death increased with systolic pressure (*P* = 0.0013) on admission [31]. In a Nigerian teaching hospital in Enugu, the case-fatality rate among patients admitted because of hypertension was 42.9% [32].

Undoubtedly, cost containment is important in the management of a common disease, such as hypertension, especially in resource poor settings, where out-of-pocket medical expenditure is usual practice. The basic problem in the countries in which NOAAH is running, is that many cheap generics are being sold with minimal quality requirements or even without any regulation via illegal channels. The situation is quite different from that in South Africa, where very reputable pharmaceutical companies from India, which have won US Food and Drug Administration (FDA) approval, are marketing high-quality generic drugs at a considerably lower cost than the branded equivalents. While the older drugs are cheaper, in the long run they contribute to the development of side effects such as the metabolic syndrome, diabetes mellitus, gout and dyslipidemia. More importantly, treatment with newer drugs results in lower risk of morbidity and mortality [7]. The final NOAAH report will include an economic analysis, but cannot address cost-benefit in terms of hard cardiovascular outcomes, because blood pressure control is the main outcome in this trial with limited sample size and short follow-up.

By design, the NOAAH trial closely follows the ESC/ESH [11] and several African [33-35] guidelines. First, NOAAH patients have uncomplicated grade-1 or grade-2 hypertension with no more than two additional risk factors. For such patients, the guidelines [11,33-35] propose that lifestyle changes should first be recommended and reinforced for several weeks (grade 2) or even months (grade 1) before antihypertensive drug treatment is initiated. Second, as recommended by most current guidelines [6,11,33-38], combination therapy is used to initiate antihypertensive treatment in NOAAH patients. Initiation of treatment with single-pill combinations is particularly indicated when the blood pressure is markedly above the hypertension threshold (more than 20 mmHg systolic or 10 mmHg diastolic), or in the presence of milder degrees of blood pressure elevation in high-risk patients. Anticipated advantages of this approach include tighter and earlier blood pressure control, simplification of the therapeutic regimen and therefore better adherence; avoidance of dose dependent adverse effects experienced with higher doses of single agents and attenuation of adverse effects of some agents when used alone. The control of blood pressure observed so far in NOAAH for the two treatment groups combined and the low rate of adverse events leading to withdrawal seem to confirm these observations. Third, according to the ABCD algorithm [39,40] both treatment arms of NOAAH include a drug class that addresses the low-renin volume component of hypertension (hydrochlorothiazide and amlodipine) as well as agents (bisoprolol and valsartan) interfering with the high-renin vasoconstrictor component [40]. Diuretics and calcium channel blockers potentiate the efficacy of renin system inhibitors in Black low-renin patients [41].

The lower-dose reference medication in NOAAH is the combination of bisoprolol 5 mg plus hydrochlorothiazide 6.25 mg. Given once daily, this combination lowers blood pressure throughout 24 hours and does so significantly more than bisoprolol 5 mg/day or hydrochlorothiazide 25 mg/day given as single components [42]. A double-blind parallel group dose escalation trial involved 539 patients with uncomplicated mild-to-moderate hypertension, who were randomized to the combination of bisoprolol plus hydrochlorothiazide (2.5/6.25, 5/6.25 and 10/6.25 mg/day, enalapril (5, 10, 20 mg/day) or amlodipine (2.5, 5, 10 mg/day) for a period of 12 weeks [43]. The combination was at least as effective as amlodipine and more effective than enalapril [43]. These findings were consistent in African Americans [44].

The present study must be interpreted within the context of its limitations. First, the current report originates from a quality control program that was put in place to monitor the clinical sites and to motivate the NOAAH investigators. Second, the dose of hydrochlorothiazide in the reference group is lower than has been used in positive outcome trials. In the comparison of the blood pressure lowering effects, this might favor the valsartan plus amlodipine combination over the combination of bisoprolol plus hydrochlorothiazide. Novartis made Exforge® available as 160 mg valsartan plus 5 mg or 10 mg amlodipine. SCC had to purchase the reference drug on the Belgian market. The only combination of older drugs, in which the dosage of one component remained constant over the dosing range (as valsartan in Exforge®) was Lodoz®: 6.25 mg hydrochlorothiazide plus 5 mg or 10 mg bisoprolol. In single-pill combinations, both components potentiate one another. Lodoz® (Ziac® on the US market) is FDA approved for the treatment of hypertension (http://www.accessdata.fda.gov/drugsatfda_docs/label/2011/020186s027s028lbl.pdf) in Blacks and non-Blacks. Lodoz® is also on the market in the African countries,where NOAAH is running. Third, clinical research in developing countries differs in several ways from that in developed countries, partly because of the cultural differences, relatively poor health care and research infrastructure, wide socioeconomic divide within the society, low literacy level of the patients, and lack of sufficient numbers of trained investigators and support personnel in these countries [45]. For these reasons, we did not implement self-measurement of blood pressure at home. We also organized three investigators’ meetings to overcome these difficulties and to familiarize the clinical researchers with all aspects of the trial. The first meeting took place in Paris, France, on 18 December 2008 with a follow-up meeting in Abuja, Nigeria, on 26 September 2009. A training workshop was organized in Douala, Cameroon, on 26–27 August 2010. It took almost two years to obtain the required approvals, to mount the required infrastructure and to train the clinical investigators. Patient recruitment started on 1 September 2010. The procedures implemented for the trial both peripherally at clinical sites and centrally at the coordinating office worked well. Randomization resulted in similar characteristics in the two treatment groups. More than half of the randomized patients were women, which will support the generalizability of the NOAAH results. At the time of submission of this progress report, enrollment was completed and 180 randomized patients were in follow-up. Finally, for the present analysis, we pooled both treatment groups and we used simple statistics. However, to increase power, the statistical analysis of the NOAAH trial, once completed, will be based on mixed models, which allow accounting for clustering of repeated measures within a patient and for randomly missing data.

## Conclusions

Current guidelines support the use of combination therapy as first-line treatment in hypertensive patients. However, to our knowledge, there is presently little information on the blood pressure lowering efficacy and the rate of adverse events on single-pill combinations consisting of older drugs as compared with newer drugs in Black hypertensive patients born and living in sub-Saharan Africa. This progress report on the NOAAH trial demonstrated that blood pressure control can be achieved fast in Black African patients with a simple regimen consisting of a single-pill combination of two antihypertensive agents. It also shows that randomized clinical trials of cardiovascular drugs can be conducted among the indigenous populations of sub-Saharan Africa by African investigators. The skills being learned by local investigators will be useful for future more demanding clinical trials.

## Appendix

### NOAAH centers

Yaoundé, Cameroon: HJ Boombhi, S Kingue; Douala, Cameroon: MM Kamdem, JS Kaptue, D Lemogoum; Libreville, Gabon: E Ecke Nzengue, JB Mipinda; Abidjan, Ivory Coast: M Adoh Adoh, E Ake-Traboulsi; Ilorin, Nigeria: A Aderibigbe, PM Kolo, BA Omotoso; Enugu, Nigeria: BC Anisiuba, B Ezeala-Adikaibe, CK Ijoma, E Shu, II Ulasi; Dakar, Senegal*:* SA Ba, MB Ndiaye.

### Organization and coordination

Sponsor: JR M’Buyamba-Kabangu, S Mampunza Ma Miezi (University of Kinshasa, Kinshasa, Democratic Republic of Congo); Coordinating Center: K Asayama, B Ezeala-Adikaibe, S Covens, Y Jin, T Kuznetsova, Y Liu, AN Odili, JA Staessen, L Thijs, S Zuba (University of Leuven, Leuven, Belgium).

### Logistics and financial support

Novartis AG (Basel, Switzerland) provided non-binding financial support and the Exforge® study medication. S Vancayzeele (Vilvoorde, Belgium), N Crétin (Dakar, Senegal), and O Nwaiwu (Lagos, Nigeria) organized logistic support. AtCor Medical Pty. Ltd. (West Ryde, New South Wales, Australia) made four SphygmoCor devices available at no cost for use in NOAAH. The European Union (grants IC15-CT98-0329-EPOGH, LSHM-CT-2006-037093 InGenious HyperCare, HEALTH-F4-2007-201550 HyperGenes and HEALTH-F7-2011- 278249 EU-MASCARA), the Fonds voor Wetenschappelijk Onderzoek Vlaanderen, Ministry of the Flemish Community, Brussels, Belgium (grants G.0575.06 and G.0734.09), and the Katholieke Universiteit Leuven, Belgium (grants OT/00/25 and OT/05/49) supported the Studies Coordinating Centre (Leuven, Belgium).

## Misc

on behalf of the Newer versus Older Antihypertensive Agents in African Hypertensive Patients (NOAAH) investigators

## Competing interest

The authors declare that they have no competing interests.

## Authors’ contributions

SK, SAB, DL, and IIU are the principal investigators at the clinical centers. BCA, MMK, MBN, CKI, JK, HJB, PMK, ENS and IIU examined patients and collected the clinical data. JRM’BK represents the sponsor of the trial and did local audits. JAS designed the protocol, obtained funding and was responsible for the day-to-day coordination of the trial. LT constructed the database. ANO and BEA contributed to database management during a three-month fellowship at the coordinating office in Leuven, Belgium. ANO, LT and JAS wrote the first draft of the manuscript. LT did the statistical analysis. All authors read and approved the final manuscript.

## Role of the funding source

Novartis provided unrestricted financial support and the Exforge study medication. The funding source had no role in the design of the trial, data collection, database management, statistical analysis, or writing of this report. The sponsor (JR M’Buyamba-Kabangu) and the scientific coordinator (JA Staessen) had full access to all the data and accept the final responsibility for the decision to submit this manuscript for publication.
